# Phloretin Protects Macrophages from *E. coli*-Induced Inflammation through the TLR4 Signaling Pathway

**DOI:** 10.4014/jmb.1910.10063

**Published:** 2019-12-30

**Authors:** Anil Kumar Chauhan, Mihee Jang, Yangmee Kim

**Affiliations:** Department of Bioscience and Biotechnology, Konkuk University, Seoul 05029, Republic of Korea

**Keywords:** Phloretin, *E. coli* infection, macrophages, TLR4 pathway, inflammation

## Abstract

Macrophages are the cells of the first-line defense system, which protect the body from foreign invaders such as bacteria. However, Gram-negative bacteria have always been the major challenge for macrophages due to the presence of lipopolysaccharides on their outer cell membrane. In the present study, we evaluated the effect of phloretin, a flavonoid commonly found in apple, on the protection of macrophages from *Escherichia coli* infection. RAW 264.7 cells infected with standard *E. coli*, or virulent *E. coli* K1 strain were treated with phloretin in a dose-dependent manner to examine its efficacy in protection of macrophages. Our results revealed that phloretin treatment reduced the production of nitric oxide (NO) and generation of reactive oxygen species along with reducing the secretion of proinflammatory cytokines induced by the *E. coli* and *E. coli* K1 strains in a concentration-dependent manner. Additionally, treatment of phloretin downregulated the expression of *E. coli*-induced major inflammatory markers i.e. cyclooxygenase-2 (COX-2) and hemeoxygenase-1 (HO-1), in a concentration dependent manner. Moreover, the TLR4-mediated NF-κB pathway was activated in *E. coli*-infected macrophages but was potentially downregulated by phloretin at the transcriptional and translational levels. Collectively, our data suggest that phloretin treatment protects macrophages from infection of virulent *E. coli* K1 strain by downregulating the TLR4-mediated signaling pathway and inhibiting NO and cytokine production, eventually protecting macrophages from *E. coli*-induced inflammation.

## Introduction

Inflammation is an immune response of the body toward foreign invaders such as bacteria, viruses, and/ or their products [1]. However, if it persists for a long time, it may cause severe life-threatening diseases such as sepsis, cardiovascular diseases, and inflammatory bowel disease [2, 3]. Macrophages, cells of the first-line innate immune defense system, are the major cells involved in inflammation [4]. The principal role of macrophages is phagocytosis through which they process microbes and generate pro-inflammatory cytokines and antimicrobial mediators to eliminate pathogens [5]. However, occasionally due to severe infection, macrophages fail to execute phagocytosis, a condition commonly known as chronic granulomatous disease where infected macrophages are aggregated in response to microbes and form a granuloma [6]. Therefore, ensuring the proper functioning of macrophages is crucial and is of upmost importance.

Natural compounds derived from edible plants and medicinal crops have gained massive attention against clinical disorders nowadays due to their less toxicity and availability in foods consumed daily [7]. Phloretin (3-(4-hydroxyphenyl)-1-(2,4,6 trihydroxy phenyl) propan-1-one) is one of the most widely studied compounds among natural compounds. It is found abundantly in apples and many plants including *Pieris japonica*, *Hoveniae lignum*, and *Loise-leuria procumbens* [8, 9]. It has been studied comprehensively due to its multifunctional roles including antioxidant [10], anti-inflammatory [11], cardioprotective [12], anticancer [13], and antibacterial activities [14]. In our recent study, we observed that phloretin has an anti-bacterial activity against *Propionibacterium acnes* bacteria [15] and it protected the HEK293 cells from the *P. acnes*-induced inflammation by specifically targeting the dimerization of TLR2/1 suggesting its potential as an antibacterial as well as anti-inflammatory agent [16].

Phloretin has been reported to have potent antibacterial activity against both gram-positive and gram-negative bacteria. Specifically, phloretin can alter the activity of key enzymes that are responsible for energy metabolism and redox balance in bacteria along with reducing the ability of bacteria to cope with oxidative stress [14]. Moreover, in our previous study, we reported that phloretin showed weak antibacterial activity against *E. coli* and *S. aureus* along with its efficacy to selectively inhibit the β-ketoacyl-acyl carrier protein synthase III (KAS III) enzyme, which is involved in fatty acid synthesis in bacteria while other flavonoids did not show antibacterial activity against *E. coli* at all [17]. Additionally, in another study, we revealed that phloretin exerts anti-tuberculosis activity against various H37Rv, multi-drug-, and extensively drug-resistant clinical isolates of *Mycobacterium tuberculosis* [18]. Further, we discovered that phloretin targets the *M. tuberculosis* KAS III (mtKAS III) enzyme, which is essential for the synthesis of mycolic acid in *M. tuberculosis*, along with protecting lung cells from interferon-γ-induced inflammation [18].

Phloretin does not only exert an antibacterial effect but can also prevent biofilm production in *E. coli* O157:H7 [19]. Treatment with phloretin causes the repression of toxin genes (*hlyE* and *stx2*), autoinducer-2 importer genes (lsrACDBF), curli genes (*csgA* and *csgB*), and many other prophage genes in *E. coli* O157:H7 biofilm cells [19]. Further, phloretin can efficiently reduce the attachment of *E. coli* to human colon epithelial cells, eventually preventing colon inflammation in a rat model [19]. Another study on inflammatory gene profiling in immunorelevant human cell lines (DLD-1, T84, MonoMac6, and Jurkat) revealed that phloretin treatment potentially downregulates inflammatory genes that are highly expressed in colon inflammation, thus protecting against inflammatory bowel disease [20].

Although the antibacterial efficacy of phloretin is well-established, in the present study, we for the first time, to the best of our knowledge, aimed to investigate the effect of phloretin in protection of macrophage from standard *E. coli* and virulent *E. coli* K1 infection. and inflammation in an *ex vivo* model. The virulent *E. coli* K1 strain RS218 (O18:K1:H7) possesses the K1 capsular polysaccharide antigen, which is essential virulence determinant that protects the bacteria from immune attack which provide additional virulence to the bacteria [21]. Results of our study might provide new insights for exploring phloretin efficacy towards infection and modulation of innate immune response.

## Materials and Methods

### Chemicals and Antibodies

All chemicals, unless otherwise stated, were of the highest quality and were used as supplied. Phloretin (with ≥99% purity) was purchased from Sigma-Aldrich (USA). Purity of phloretin was confirmed by HPLC and Mass spectrometer (KBSI). Anti- myeloid differentiation primary response 88 (MyD88), phosphorylation of transforming growth factor beta-activated kinase 1 (p-TAK1), antibodies were purchased from Abcam (UK). Anti-cyclooxygenase-2 (COX-2), hemeoxygenase-1 (HO-1), Actin, and p-NF-κB antibodies were purchased from Cell Signaling (USA).

### Cells and Bacterial Culture

RAW 264.7, a murine macrophage cell line, was purchased from the American Type Culture Collection (Manassas, VA) and cultured in Roswell Park Memorial Institute (RPMI) 1640 medium (Invitrogen, USA) supplemented with 10% (v/v) fetal calf serum (Invitrogen,) and 1% penicillin–streptomycin cocktail at 37°C in a 5% CO_2_ incubator.

*E. coli* (KCTC 1682) was purchased from the Korean Collection for Type Cultures, Korea Research Institute of Bioscience & Biotechnology (Korea). *E. coli* K1 strain RS218 (O18:K1:H7), was provided by Jang-Won Yoon of Kangwon National University (Korea). The cultures were maintained in Luria Bertani broth/agar.

### RAW 264.7 Cell Infection Model

Infection of RAW 264.7 cells with *E. coli* was performed as described previously [22] with minor modifications. Briefly, RAW 264.7 cells were seeded in a 6-well plate and incubated at 37°C in a CO_2_ incubator for 24 h, after which they were infected with overnight-grown standard *E. coli* and virulent *E. coli* K1 at a multiplicity of infection (MOI) of 10 for 1 h. Infected cells were then treated with phloretin (50, 100, and 150 mM), and the plates were incubated at 37°C in a CO_2_ incubator for 16 h.

### Detection of Nitric Oxide (NO)

Cells were infected with overnight-grown standard *E. coli* and virulent *E. coli* K1 at a multiplicity of infection (MOI) of 10 for 1 h. Infected cells were then treated with phloretin (50, 100, and 150 μM), and the plates were incubated at 37°C in a CO_2_ incubator for 16 h. Secretion of NO was observed in cell supernatant by using the modified Griess reagent (Sigma-Aldrich), and examination was carried out as described by the manufacturer.

### Detection of Reactive Oxygen Species (ROS)

ROS were detected using 2’,7’-Dichlorofluorescin diacetate (Sigma-Aldrich). Cells were infected and treated with phloretin as described above, and thereafter, the cell medium was removed and cells were washed with phosphate buffered saline (PBS), and then 25 μM of ROS stain was added to the cells for 30 min. Thereafter, cells were washed twice with PBS, and then the cell suspension was collected. The fluorescence intensity was measured using a fluorometer.

### Detection of Cytokine Secretion

To examine cytokine (TNF-α and IL-6) secretion, we collected the supernatant of the non- infected, infected, and phloretin-treated groups, and then TNF-α and IL-6 were examined using ELISA kits (Invivogen, USA) according to the manufacturer’s instructions.

### Immunoblot Analysis

For western blot analysis, an equal amount of protein lysate (50 μg in each lane) was mixed with 5× sample buffer (50 mM Tris of pH 6.8, 2% SDS, 10% glycerol, 1% DTT, and 0.1% bromophenol blue) and heated for 5 min at 95oC, followed by SDS–polyacrylamide gel electrophoresis. After electrophoresis, samples were transferred to a polyvinylidene fluoride (PVDF) membrane (Roche Diagnostics, USA) by electroplating. The blots were probed with primary antibodies, followed by horseradish peroxidase-conjugated secondary antibody, and then visualized by enhanced chemiluminescence (ECL) according to the recommended procedure (Amersham Pharmacia, USA).

### RT-PCR Examination

TLR4 pathway gene expression was examined using RT-PCR analysis. Briefly, RAW264.7 cells were infected with overnight-grown virulent *E. coli* K1 at a multiplicity of infection (MOI) of 10 for 1 h. Infected cells were then treated with phloretin (50, 100, and 150 μM), and the plates were incubated at 37°C in a CO_2_ incubator for 16 h. Thereafter, RNA was isolated using Tri-RNA Reagent (Favorgen, Taiwan) and cDNA synthesis was carried out by using a SuperScript III Reverse Transcriptase kit (Thermo Scientific, USA). Thereafter, the synthesized cDNA was used as a template for the PCR reaction against TLR4, MyD88, and NF-κB primers. GAPDH was used as the internal control (primer sequences are listed in Table 1). Eventually, the obtained PCR product was resolved in 1.5% agarose gel and the images were captured after staining with ethidium bromide.

### Statistical Analysis

Experimental results are depicted as the mean ± standard error of the mean (SEM) of three individual sets of experiments. Statistical significance was calculated using the Student’s *t*-test. *represents *p* < 0.05, **represents *p* <0.01, and ***represents *p* <0.001.

## Results

### Phloretin Treatment Reduced the Secretion of NO and Generation of ROS

**Fig. 1:**
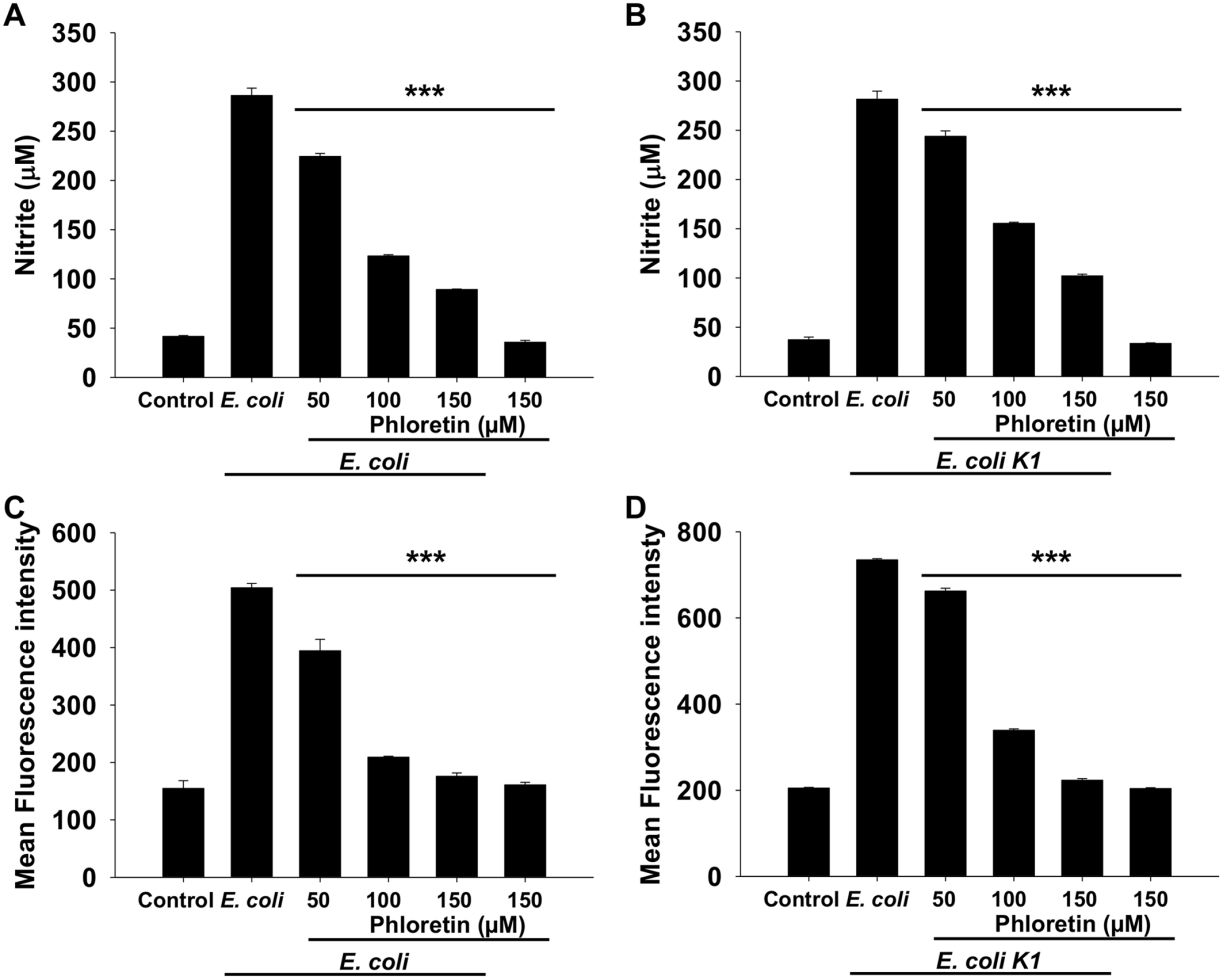
Effect of phloretin on the reduction of NO and ROS level in RAW264.7 cells infected with standard *E. coli* and *E. coli* K1. (**A**) Nitric oxide level in RAW264.7 cells infected with standard *E. coli*. (**B**) Intensity of ROS in RAW264.7 cells infected with *E. coli* K1. (**C**) Nitric oxide level in RAW264.7 cells infected with standard *E. coli*. (**D**) Intensity of ROS in RAW264.7 cells infected with *E. coli* K1. Data are presented as means ± SEMs. *, *p* < 0.05; **, *p* < 0.01; and ***, *p* < 0.001 compared with the *E. coli* infected group.

The effect of phloretin on secretion of NO and generation of ROS was observed in RAW264.7 cells in both standard and virulent *E. coli* strains. As depicted in Fig. 1, treatment with phloretin significantly reduced the secretion of NO in RAW264.7 cells induced by both the standard and virulent *E. coli* strains compared to that in *E. coli* only-infected cells. NO secretion levels of 224.4, 123.3, and 89.28 μM were observed in standard *E. coli* strain-infected cells, while the secretion levels observed were 243.63, 155.5, and 102.1 μM in *E. coli* K1 strain-infected cells at phloretin concentrations of 50, 100, and 150 μM, respectively. Contrastingly, NO concentrations in *E. coli* and *E. coli* K1 strain-infected cells were 286.2 and 281.63 μM, respectively, suggesting that phloretin was effective in reducing NO levels in both strains (Fig. 1A and 1B). Similarly, the generation of intracellular ROS was also significantly reduced after treatment with phloretin in a concentration-dependent manner. For instance, the intensities of ROS generation in *E. coli* and *E. coli* K1 strain-infected cells were significantly higher than control, however, at phloretin concentrations of 50, 100, and 150 μM, the intensities of ROS were reduced up to 21, 58, and 65% respectively against standard *E. coli* and 9, 53, and 69% against virulent *E. coli* K1 strains, respectively (Fig. 1C and 1D). These results suggest that infection of *E. coli* and *E. coli K1* strain induced the RAW264.7 cells to produce NO and intracellular ROS in order to generate inflammation and oxidative stress. However, treatment with phloretin protected macrophages from *E. coli* infection-induced inflammation and oxidative stress.

### Secretion of Proinflammatory Cytokines Was Decreased by Phloretin Treatment

**Fig. 2:**
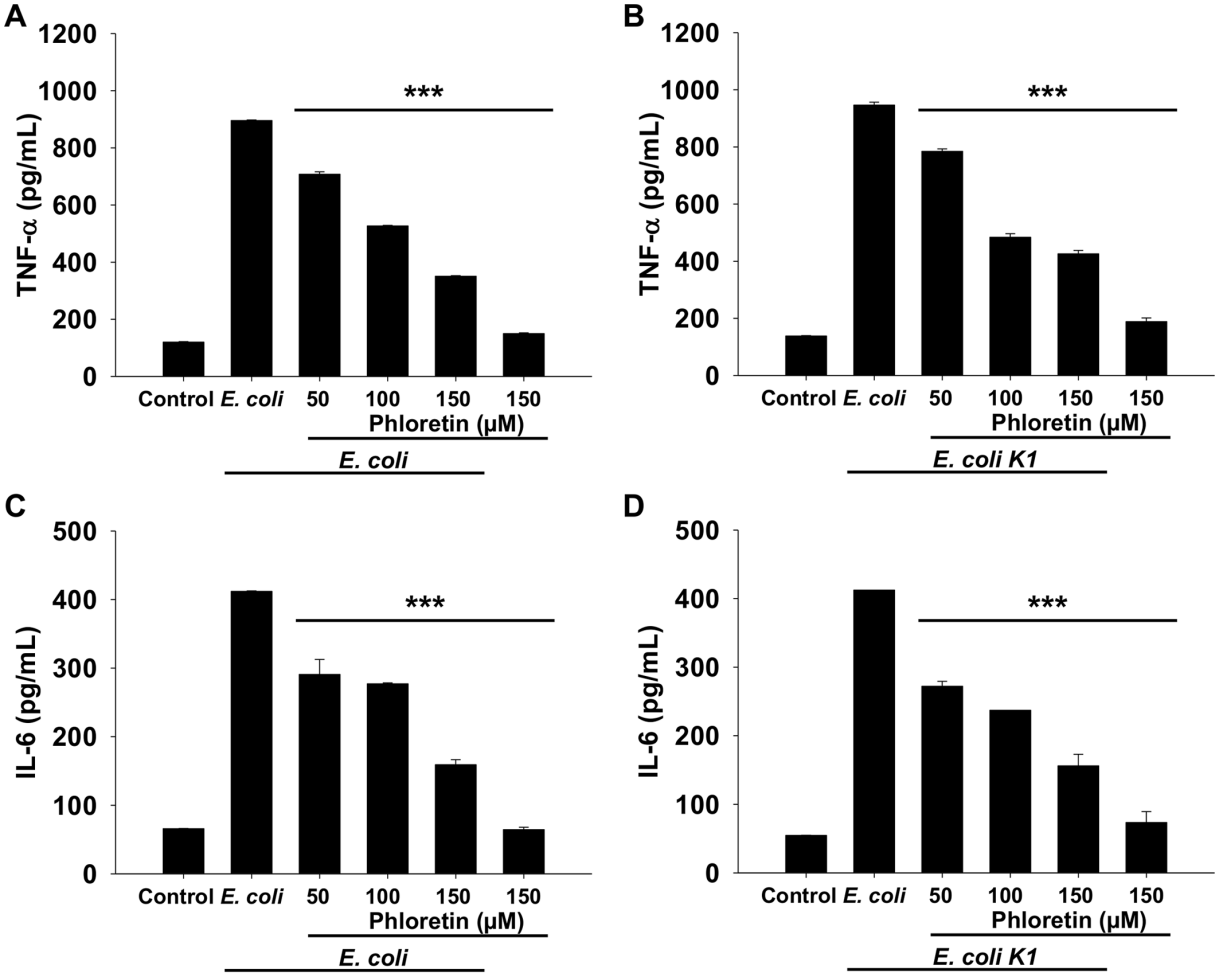
Effect of phloretin on cytokine secretion in RAW264.7 cells infected with standard *E. coli* and *E. coli* K1. (**A**) Level of TNF-α in RAW264.7 cells infected with standard *E. coli*. (**B**) Level of TNF-α in RAW264.7 cells infected with *E. coli* K1. (**C**) Level of IL- 6 in RAW264.7 cells infected with standard *E. coli*. (**D**) Level of IL-6 in RAW264.7 cells infected with *E. coli* K1. Data are presented as means ± SEMs. *, *p* < 0.05; **, *p* < 0.01; and ***, *p* < 0.001 compared with the *E. coli* infected group.

We observed that treatment with phloretin decreased the production of NO, which is directly associated with inflammation; therefore, we observed the effect of phloretin on the secretion of TNF-α and IL-6, the major proinflammatory cytokines. As depicted in Fig. 2A andacrophages infected with standard *E. coli* tended to produce a high amount of TNF-α and IL-6 however, treatment with phloretin caused a significant reduction in the secretion of both cytokines in a concentration-dependent manner. At a concentration of 100 μM, the TNF-α and IL-6 levels decreased by 41.17% and 42.5%, respectively, compared to those in the *E. coli*-treated group.

Similarly, infection of macrophages with *E. coli* K1 also induced the secretion of large amounts of TNF-α and IL-6 (Fig. 2C and 2D). In contrast, phloretin treatment (50, 100, and 150 μM) significantly decreased the secretion level of TNF-α and IL-6. All the concentrations used were effective in reducing cytokine secretion, with the 100 μM concentration reducing the secretion of TNF-α by 48.9% and that of IL-6 by 32.7% in comparison to that in the *E. coli* K1-treated group (Fig. 2C and 2D), suggesting the potent anti-inflammatory potential of phloretin.

### Phloretin Targets the TLR4-Induced NF-κb Pathway to Protect Macrophages from *E. coli* Infection

**Fig. 3:**
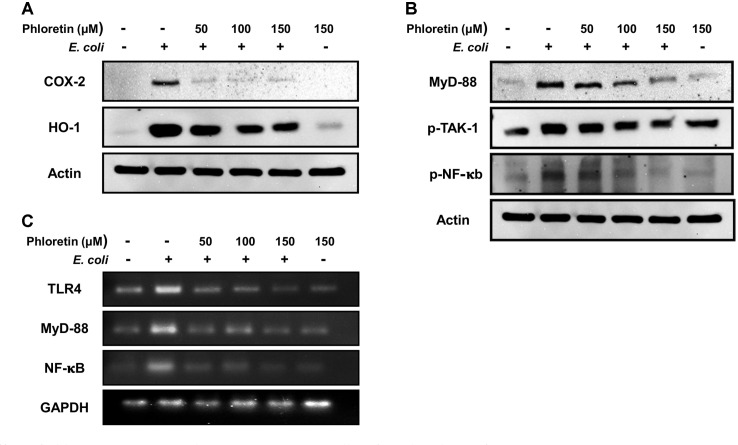
Effect of phloretin on TLR4 pathway in RAW264.7 cells infected with *E. coli* K1. (**A**) Effect of phloretin on the expression of HO-1 and COX-2 examined by immunoblotting. (**B**) Effect of phloretin on the expression of MyD88, p- TAK-1, and p-NF-κb proteins examined by immunoblotting. (**C**) Effect of phloretin on the expression of TLR4, MyD88, and NF-κB genes examined by RT-PCR.

We observed that phloretin potentially reduced RAW264.7 cell inflammation induced by standard *E. coli* and *E. coli* K1 strains. Therefore, we next sought to examine the mechanism underlying the anti-inflammatory activity of phloretin. A previous report has suggested that *E. coli* targets the TLR4 pathway to induce inflammation via lipid A of the lipopolysaccharide (LPS) found in its cell wall [23]. Thus, we performed immunoblotting and RT-PCR analysis to examine the TLR4- NF-κb pathway. As an initial step, we examined the major inflammatory markers, HO-1 and COX-2, which are highly expressed during inflammation. As depicted in Fig. 3A, infection of macrophages with *E. coli* K1 induced the up-regulation of HO-1 and COX-2 proteins, which were highly downregulated in phloretin-treated cells (Fig. 3A), suggesting that phloretin protects the cells from inflammation.

We also examined the expression pattern of myeloid differentiation primary response 88 (MyD88), phosphory-lation of transforming growth factor beta-activated kinase 1 (p-TAK1), and phosphorylation of NF-κb proteins, which are associated with the production of cytokines to induce inflammation. Results revealed that cells infected with *E. coli* K1 exhibited up-regulation of MyD88 and phosphorylation of TAK-1, which were significantly decreased in the phloretin-treated group in a concentration-dependent manner (Fig. 3B). Moreover, *E. coli* K1 infection caused the phosphorylation of NF-κb, which was potentially inhibited after phloretin treatment, suggesting that phloretin treatment inhibited the translocation of NF-κb from the cytosol to the nucleus (Fig. 3B). We further evaluated the expression of TLR4, MyD88, and NF-κb at the transcriptional level by RT-PCR. As depicted in Fig. 3C, all the proteins were highly upregulated in the *E. coli* K1-infected group, but were downregulated in the phloretin-treated group in a concentration-dependent manner (Fig. 3C). These results suggest that phloretin targeted the TLR4-NF-κb pathway to suppress *E. coli* K1-induced inflammation in RAW264.7 cells.

## Discussion

*E. coli* is a gram negative, flagellar bacterium belonging to the family Enterobacteriaceae and has been widely studied in microbiology [24]. The pathogenicity of different *E. coli* strains depends on the antigen found on their surface i.e. somatic (O antigen), capsular (K antigen) and flagellar (H antigen) based on which serotyping of *E. coli* has been done [25]. Moreover, based on the serotyping and their ability to cause the disease, *E. coli* has been divided in several groups, for example diarrhoeagenic *E. coli* (DEC), uropathogenic *E. coli* (UPEC), septicaemic *E. coli* (SePEC), neonatal meningitis-associated *E. coli* (NMEC) [25, 26].

Urinary tract infection and kidney damage are the most common infections caused by *E. coli* strains [27]; however, they are also reported to cause meningitis, Crohn’s disease, and sepsis, the deadliest disease [28, 29]. The *E. coli* K1 strain have been reported to be the most virulent strain due to the K1 capsular polysaccharide which helps them to bind with human brain microvascular endothelial cells (HBMECs) and crossing the blood brain barrier [30]. Also, with unclear mechanism it has been observed that most of these K1 isolates are associated with a limited number of O serotypes (*e.g.*, O18, O7, O16, O1, O45) [30] which enhances their virulence. On the other hand, humans have a sophisticated innate immune system that deals with extraneous invaders wherein macrophages play a crucial role by eliminating bacteria by phagocytosis [4, 5]. Therefore, the proper functioning of macrophages is essential, and using this as a basis, we designed this study to investigate the effect of phloretin in the protection of macrophage from inflammation induced by standard *E. coli* and virulent *E. coli K1*.

As an initial step, we evaluated the potential of phloretin to reduce NO production induced by *E. coli*. NO is produced by macrophages during phagocytosis to kill aerobic bacteria; however, exaggerated production of NO is associated with the induction of the inflammation pathway, and thus, NO is considered as a primary diagnostic marker of inflammatory diseases [31, 32]. In our study, we used standard *E. coli* and virulent *E. coli* K1 strains to infect RAW264.7 cells and compared the effect of phloretin in reducing NO levels. Our results suggested that treatment of cells with both bacterial strains tended to massively enhance the production of NO, an effect that was blocked by phloretin in a concentration-dependent manner. Even the lowest concentration of phloretin (50 μM) decreased the level of NO; however, the phloretin concentration of 50 μM was found to be the most effective, suggesting that phloretin treatment protects macrophages from *E. coli*-infection-induced NO production.

 Another major marker of inflammation is the production of cytokines, which are basically small secretory proteins that act as cell-to-cell communicators and are majorly of two types, namely, pro-inflammatory and anti-inflammatory cytokines [33]. Activated macrophages are responsible for the production of pro-inflammatory cytokines, which upregulate the inflammatory pathway. Among these cytokines, IL-6 and TNF-α are commonly observed to be secreted during inflammation [33]. Therefore, we examined the efficacy of phloretin against the production of these major pro-inflammatory cytokines IL-6 and TNF-α in our study. The results revealed that phloretin treatment decreased the secretion of both cytokines in a concentration-dependent manner. Contrastingly, between standard *E. coli*- and virulent *E. coli* K1-infected cells, *E. coli* K1-infected cells tended to produce a higher amount of IL-6 and TNF-α. Therefore, we suggest that phloretin can protect macrophages from inflammation even if they are infected with a virulent *E. coli* strain.

The molecular mechanism of inflammation has been well-elucidated, and a previous report suggested that activation of the TLR4 pathway is the most common event in fighting against gram-negative bacteria [23, 34]. Upon the interaction of macrophages with gram negative bacteria, the lipid portion of LPS found in the outer layer of gram-negative bacteria binds to MD2, which causes the activation of the TLR4 pathway by dimerization of TLR4 protein with MD2 [23]. Once TLR4 is activated, MyD88 is overexpressed, resulting in the phosphorylation of TAK-1 protein, and this phosphorylation results in IKKb phos-phorylation, which eventually causes NF-κb phosphorylation and translocation from the cytosol to the nucleus to produce cytokines [35]. We, therefore, investigated the ability of phloretin to regulate the TLR4-NF-κb pathway through immunoblotting and RT-PCR. Immunoblotting data revealed that phloretin downregulated the MyD88 protein along with preventing TAK-1 and NF-κb phosphorylation, which were highly up-regulated in *E. coli* K1-treated cells. Similarly, a massive downregulation of these proteins at the transcriptional level, as well as TLR4 protein, was observed in RT-PCR analysis, suggesting that phloretin might inhibit the expression of the TLR4 pathway to protect macrophages from inflammation.

In conclusion, data obtained from our study advocate the efficacy of phloretin to protect macrophages from virulent *E. coli* K1 strain infection-induced inflammation. Moreover, the ability of phloretin to protect macrophages could be attributed to its potential in downregulation of the TLR4 pathway activated by both of the *E. coli* strains. Therefore, phloretin could be an ideal candidate immunomodulator and anti-inflammatory agent against diseases caused by *E. coli* infection. However, further study using animal infection model would be needed to warrant the *ex vivo* efficacy of phloretin.

## Figures and Tables

**Table 1:** Primer sequence of TLR4 pathway genes used in RT-PCR.

Gene	Sequence	ID
TLR4	Forward-AGTGGGTCAAGGAACAGAAGCA	NM_021297.3
	Reverse-CTTTACCAGCT	
MyD88	Forward-ACGCACCTCAGTACACACAT	NM_010851.3
	Reverse-CGTGCCACTACCTGTAGCAA	
NF-κB	Forward-ATGCGCTTCCGCTACAAGTG	XM_028833232.1
	Reverse-ACAATGGCCACTTGTCGGTG	
GAPDH	Forward-AACTTTGGCATTGTGGAAGG	BC023196.2
	Reverse-ACACATTGGGGGTAGGAACA	
